# 

**Published:** 1885-04

**Authors:** 


CONTENTS.

Vol. 32 No. 4.

| | PAGE |
|---|---|
| The Sense of Taste | 1 |
| Idiosyncrasy | 2 |
| Colorless Iodine | 3 |
| Teachers' Duties | 3 |
| Kleptomania | 4 |
| Avoid the Wells | 5 |
| Cocoa | 6 |
| Mantels and Grates | 7 |
| Cholera | 9 |
| The Cosmetics of the Market | 17 |
| Cocain | 18 |
| The Therapeutics of "Horizontal Position" | 18 |
| Medical Swindlers | 18 |
| Sleep and Sound Sleepers | 20 |
| How to Make a Paper Fan | 20 |

